# Gene model-related m6A expression levels predict the risk of preeclampsia

**DOI:** 10.1186/s12920-022-01254-4

**Published:** 2022-05-05

**Authors:** Yiwei Li, Can Chen, Mengyuan Diao, Yanli Wei, Ying Zhu, Wei Hu

**Affiliations:** 1 Department of Critical Care Medicine, Affiliated Hangzhou First People’s Hospital, Zhejiang University School of Medicine, 216 Huansha Road, Hangzhou, 310006 Zhejiang China; 2 Department of Hematology, Affiliated Hangzhou First People’s Hospital, Zhejiang University School of Medicine, 216 Huansha Road, Hangzhou, 310006 Zhejiang China

**Keywords:** Preeclampsia, N6-methyladenosine, Hub gene, Prognosis, Bioinformatic gene analysis, Biomarkers

## Abstract

**Background:**

This is the first study to explore the potential functions and expression patterns of RNA N6-methyladenosine (m6A) and potential related genes in preeclampsia.

**Methods:**

We identified two m6A modification patterns through unsupervised cluster analysis and validated them by principal component analysis. We quantified the relative abundance of specific infiltrating immunocytes using single-sample gene set enrichment analysis (ssGSEA) and the Wilcoxon test. To screen hub genes related to m6A regulators, we performed weighted gene coexpression network analysis. Functional enrichment analysis was conducted for differential signalling pathways and cellular processes. Preeclampsia patients were grouped by consensus clustering based on differentially expressed hub genes and the relationship between different gene-mediated classifications and clinical features.

**Results:**

Two m6A clusters in preeclampsia, cluster A and cluster B, were determined based on the expression of 17 m6A modification regulators; ssGSEA revealed seven significantly different immune cell subtypes between the two clusters. A total of 1393 DEGs and nine potential m6A-modified hub genes were screened. We divided the patients into two groups based on the expression of these nine genes. We found that almost all the patients in m6A cluster A were classified into hub gene cluster 1 and that a lower gestational age may be associated with more m6A-associated events.

**Conclusions:**

This study revealed that hub gene-mediated classification is consistent with m6A modification clusters for predicting the clinical characteristics of patients with preeclampsia. Our results provide new insights into the molecular mechanisms of preeclampsia.

**Supplementary Information:**

The online version contains supplementary material available at 10.1186/s12920-022-01254-4.

## Background

Preeclampsia (PE) is one of the most serious hypertensive disorders of pregnancy, affecting 5–7% of all pregnant women and causing over 70,000 maternal deaths and 500,000 foetal deaths worldwide every year [1, 2]. PE presents as new-onset vascular dysfunction and may exacerbate stroke, kidney failure, pulmonary edema, liver rupture, and eclampsia during the third trimester. The exact aetiology of PE is not clear, although this is an interesting area of research. The widely held theory is that conditions such as impaired trophoblast invasion, incomplete spiral artery remodelling, endothelial dysfunction, oxidative stress and inappropriate immune responses may affect the expressed PE phenotype [3]. Nevertheless, its origin cannot be fully explained by our current understanding. In recent years, an increasing number of studies have focused on how epigenetic modification, including DNA methylation, noncoding RNA and, more marginally, histone posttranslational modification, impacts gene expression in the initiation and development of PE [4, 5]. Therefore, it is critical to explore the specific molecular mechanisms underlying the evolution of PE and identify new prognostic factors and potential therapeutic targets.

Canonical epigenetic modification research has focused on the DNA in chromatin, whereas with the deepening of research, epigenetic RNA modifications have recently begun to gain increasing attention. To date, over 160 kinds of RNA modifications have been confirmed, including N6-methyladenosine (m6A), 5-methylcytosine (m5C) and N1-methyladenosine (m1A), of which m6A modification is regarded as the most universal posttranscriptional modification in all eukaryotes, especially in determining the splicing, stability, export, decay and translation of mRNAs. Furthermore, similar to DNA and histone modification, m6A modification is dynamic and reversible in controlling gene expression; additionally, it is associated with three groups of enzymes, i.e., the “erasers” (demethylases) FTO and ALKBH5, the “readers” (binding proteins) YTHDFs and IGF2BPs, and the “writers” (RNA methyltransferases) METTL3, METTL14 and WTAP [6]. Emerging studies have shown that m6A modification and its regulators are involved in not only the pathogenesis and development of multiple diseases, including cancer [7], neurological diseases [8], infertility [9], obesity [10] and immunological diseases [11], but also the regulation of placental development and pathologies.

A previous study demonstrated that the level of global DNA methylation increased with gestational weeks, which means that gestational age may be responsible for interplacental variation and a programmed, reproducible change in DNA methylation [12]. A recent study indicated that compared to control placentas, low-birth-weight placentas were associated with significantly higher m6A expression in key genes related to lipid metabolism and angiogenesis by analysis of m6A modification levels in 44 placental samples from eight sows [10]. The study by Taniguchi revealed that m6A at the 5’-untranslated region (UTR) and near the stop codon in placental mRNA may play vital roles in foetal growth and PE [13]. Although the above discoveries provide new insights into the epigenetic mechanisms of PE, the general expression characteristics of m6A regulators in PE remain to be elucidated.

Herein, to systematically analyse the modification patterns of m6A regulators and related genes in PE, we carried out this study based on the public Gene Expression Omnibus (GEO) database. In addition, immune microenvironment infiltration characteristics were analysed. We found that m6A modification patterns were associated with some clinical features of PE and infiltration of multiple immune cells. We also revealed that m6A phenotype-related hub genes could well reflect the m6A level and be used to identify patients at high risk for PE.

## Methods

### Data acquisition

Gene expression data and the clinical data were obtained from the GEO database (https://www.ncbi.nlm.nih.gov/geo/). GSE75010 (n = 80) and GSE60438 (n = 60) were gathered in the present study for further analysis. To normalize the expression data from different datasets, the R package “sva” was used to remove batch effects [14]. A total of 21 regulators related to m6A modification were included with reference to previous studies in the follow-up analyses. These 21 m6A regulators included eight writers (METTL3, RBM15, METTL14, RBM15B, CBLL1, WTAP, KIAA1429, ZC3H13), eleven readers (YTHDF1, YTHDF2, YTHDF3, YTHDC1, YTHDC2, IGF2BP1, FMR1, HNRNPA2B1, ELAVL1, HNRNPC, LRPPRC) and two erasers (ALKBH5, FTO). Among them, 17 m6A methylase regulators were supported by expression profile data.

### Sample classification

To determine distinct m6A modification patterns in PE, we conducted unsupervised consensus clustering analysis based on the 17 m6A regulators by using the R package Consensus Cluster Plus, and the consensus clustering algorithm ran 1,000 repetitions to guarantee the stability of classification. Principal component analysis (PCA) was conducted to further verify the expression patterns of the 17 m6A regulators in different modification patterns.

### Correlation between m6A regulators and immune characteristics

To quantify the relative abundance of specific infiltrating immunocytes in each sample, a single-sample gene-set enrichment analysis (ssGSEA) algorithm was used. The Wilcoxon test was used to compare the enrichment scores among the groups.

### Identification and functional analysis of m6A-mediated genes

To identify m6A regulator-mediated genes, the empirical Bayesian approach of the ‘limma’ R package was applied to determine differentially expressed genes (DEGs) between two distinct m6A subtypes. The false discovery rate (FDR) was applied for multiple testing correction of raw P values through the Benjamini–Hochberg method. A |log2| fold change (FC)|> 1 and an FDR < 0.01 were set as the thresholds for identifying DEGs.

The R software package weighted gene coexpression network analysis (WGCNA) was used to identify the gene modules that were significantly correlated with different m6A modifications. The analysis setting included the following main steps: (1) construct the similarity matrix; (2) select the weighting coefficient, β, and transform the similarity matrix into an adjacency matrix; (3) transform the adjacency matrix into a topological overlap matrix (TOM); and (4) perform hierarchical clustering to identify modules. Finally, we selected the minimum module eigengenes (MEs) with > 25 genes and a cutting height of 0.25. Relevant modules of high importance for clinical traits were identified (Additional file 1: Table S1).

In addition, the correlations between modules and m6A modification patterns were identified by mean absolute values of gene significance (GS) through Pearson correlation. The larger the mean absolute value was, the more strongly the module was related to the m6A modification patterns.

To further study the function of these m6A-related module genes, Kyoto Encyclopedia of Genes and Genomes (KEGG) pathway analysis and Gene Ontology (GO) annotation analysis were performed using the R package ClusterProfiler through GO (http://www.geneontology.org) and KEGG database (www.kegg.jp/kegg/kegg1.html). *P* < 0.05 was chosen as the cut-off value.

### Identification of the m6A-related hub genes for classifying PE patients

We defined the genes with a GS over 0.5 and a module membership (MM) over 0.75 in the green gene module as hub genes. To classify PE RNA sequence results into different gene-mediated classifications, we used k-means unsupervised clustering analyses based on different expression patterns of nine hub genes. Through methods of fast t-distributed stochastic neighbour embedding (t-SNE) and dimension reduction, PE samples were clearly classified into different subtypes.

## Result

### Characteristics of datasets and patients

A total of 140 PE RNA sequence results were obtained from two different database (GSE75010 and GSE60438). The median age of the patients was 32 years. Sixty-two patients had a gestational age of more than 32 weeks.

**Fig. 1:**
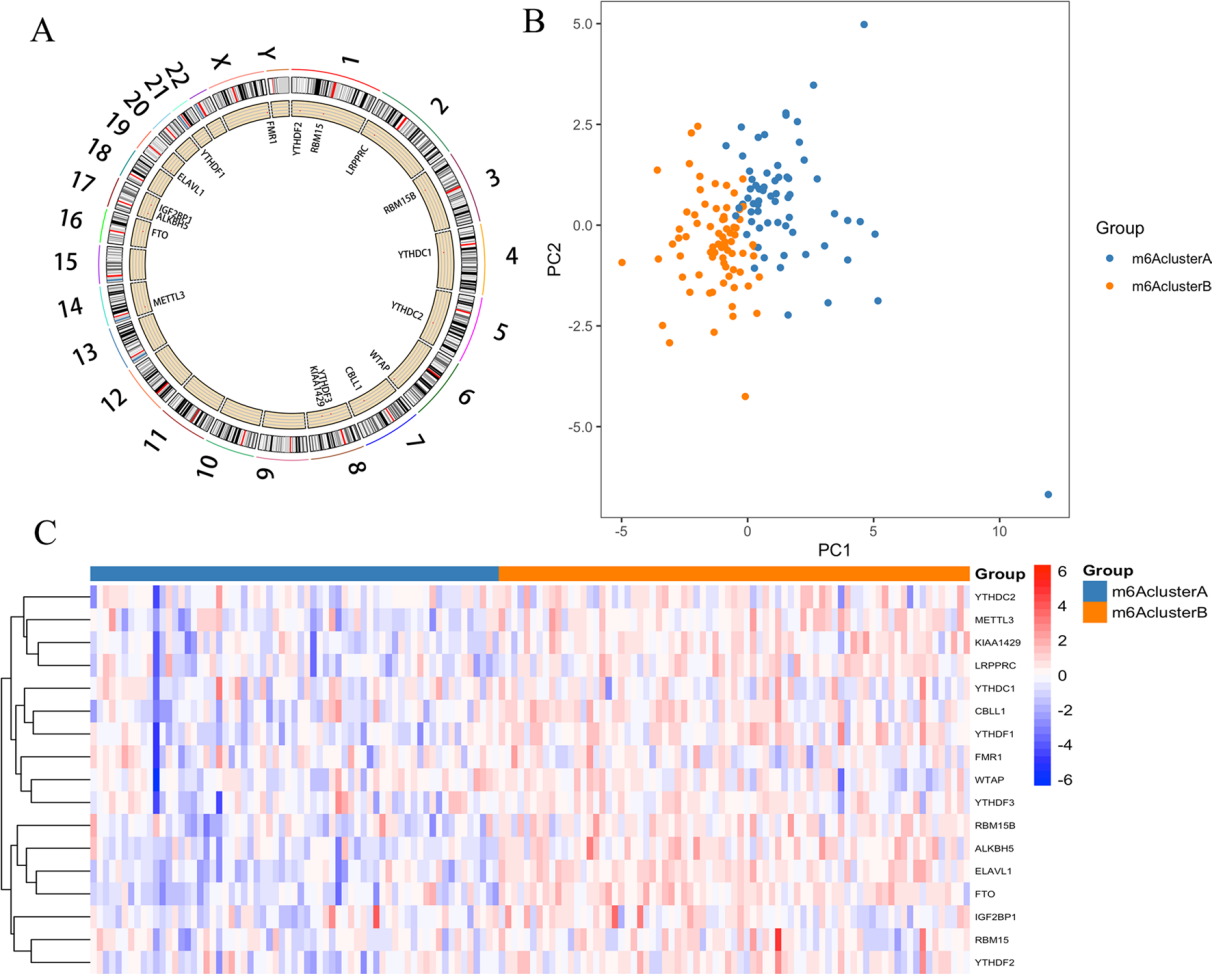
Bioinformatics analysis of the expression and genetic variation of m6A regulators in PE. **a** Overview of m6A gene locus and gene information. **b** Consensus clustering identified two subgroups according to the expression of m6A regulators. **c** Heatmap of the expression of 19 m6A regulators in two distinct m6A clusters. PE: preeclampsia

We reviewed the literature and collated a list of 21 m6A RNA methylation regulators. There were 17 m6A regulators expressed in both datasets. These 17 m6A regulators included METTL3, RBM15, RBM15B, WTAP, KIAA1429, CBLL1, ALKBH5, FTO, YTHDC1, YTHDC2, YTHDF1, YTHDF2, YTHDF3, IGF2BP1, FMR1, LRPPRC and ELAVL1. Their chromosomal locations are shown in Fig. 1a.

### m6A molecular subtypes

**Fig. 2:**
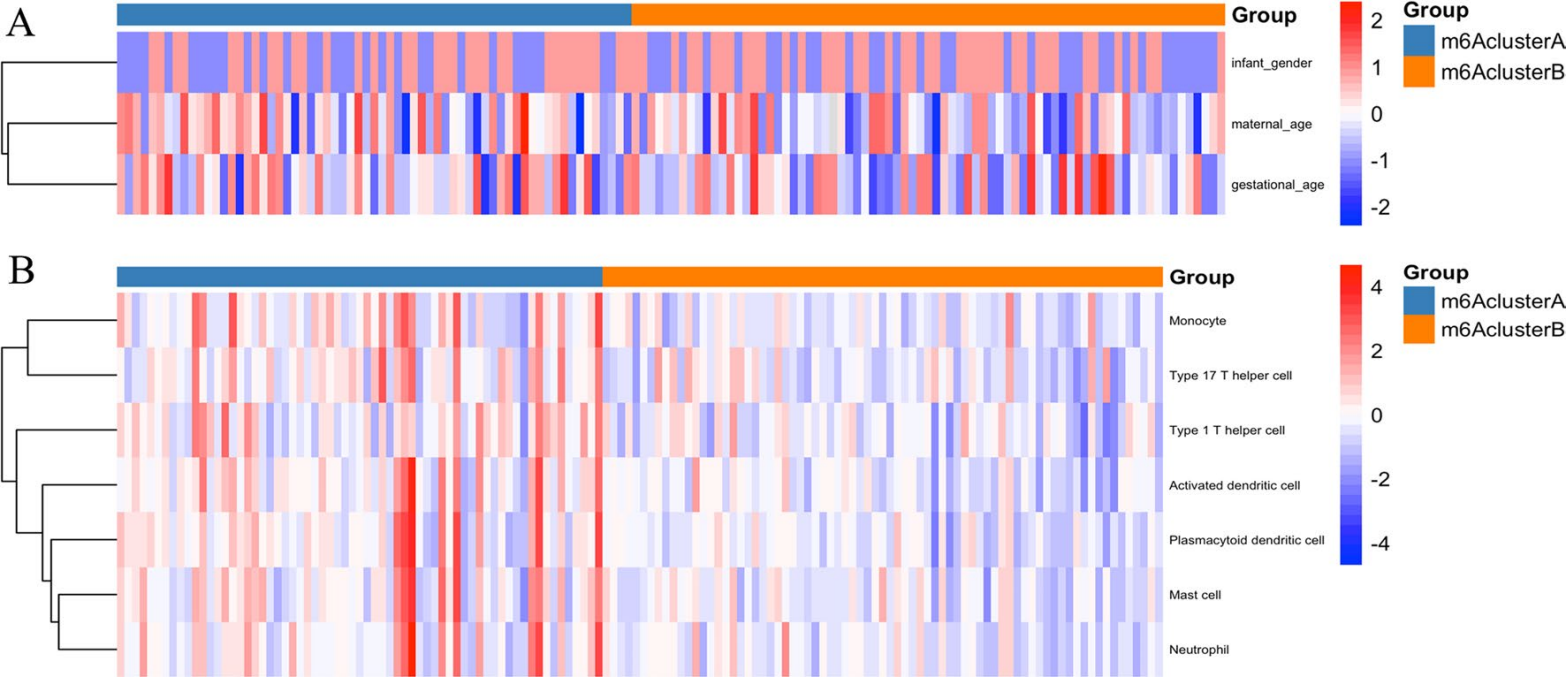
m6A methylation modification patterns are correlated with the clinical characteristics and immune cell infiltration. **a**, **b** clinicopathological features between the two clusters were compared. **c** Heatmap of immune cell infiltration in two distinct m6A clusters

According to the expression similarity of m6A RNA methylation regulators, k = 2 seemed to be an adequate selection with clustering stability increasing from k = 2 to 6 in the cohort (Additional file 2: Fig. S1, Additional file 3: Fig. S2, Additional file 4: Fig. S3). Based on unsupervised clustering, we identified two distinct modification patterns, including 65 cases in m6A cluster A and 75 cases in m6A cluster B (Fig. 1b). Most m6A RNA methylation regulator expression showed clear distinctions and significant differences in the two cluster subgroups (Fig. 1c). PE samples could be completely distinguished into two clusters based on PCA. Furthermore, the clinical features between the two subtypes were compared. Due to the limited clinical data, maternal age with a cut-off value of 32 was the only significantly different feature (*P* = 0.011) (Fig. 2a, Additional file 5: Fig. S4).

### Immune status evaluation among m6A subtypes

After ssGSEA, 28 kinds of immune cell subtypes were identified, and seven of them were significantly different between the two clusters according to the Wilcoxon test (Fig. 2b). In m6A cluster A, monocytes, activated dendritic cells, plasmacytoid dendritic cells, type 17 T helper cells, type 1 T helper cells, mast cells and neutrophils were upregulated.

### Heterogeneity of other biological processes among subtypes

**Fig. 3:**
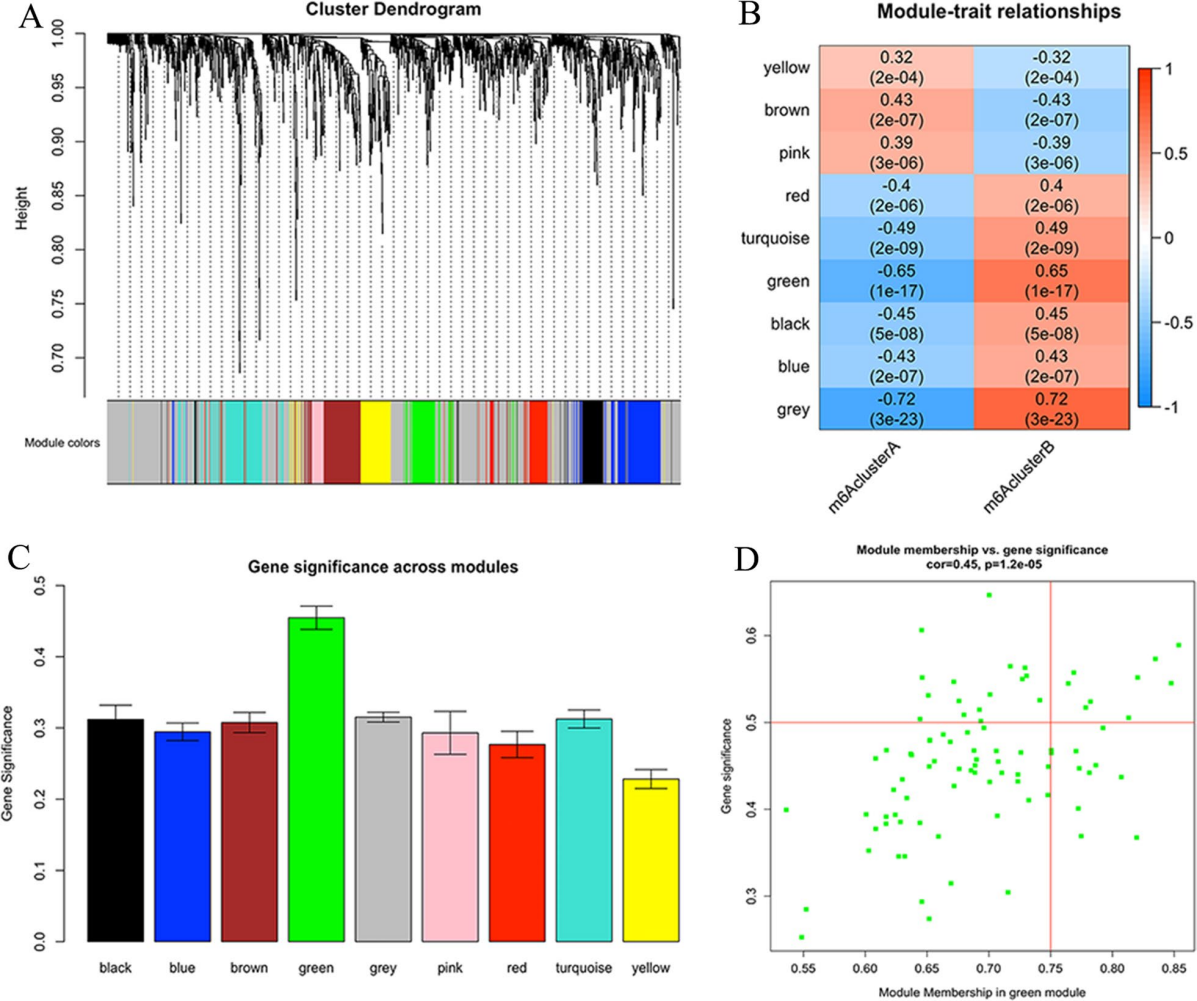
Biological characteristics of key m6A module. **a** Dendrogram of all DEGs obtained by average linkage hierarchical clustering. **b** Heatmap of the Correlation between module eigengenes and m6A modification patterns. Each cell contains the correlation coefficient and *P*-value. **c** Bar plots of mean GS across modules. We selected the ME green module for subsequent analysis. **d** Scatter plot of module eigengenes related to m6A modification patterns in the green module. DEGs: differentially expressed genes; GS: gene significance; ME: minimum module eigengene

**Fig. 4:**
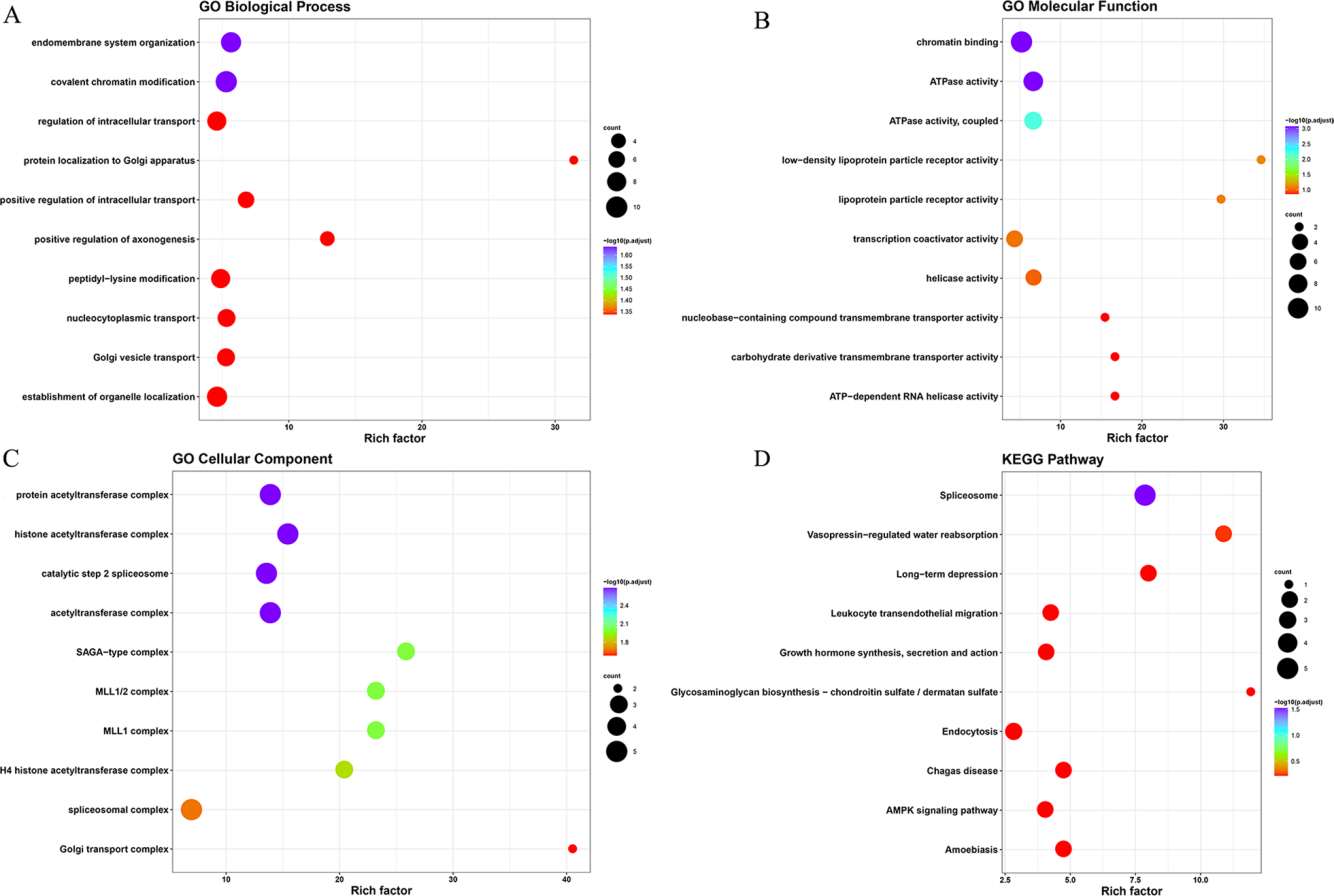
**a**–**c** Genes from green module in the biological process, molecular function, and cellular component. **d** Enrichment plot conducted via KEGG analysis. KEGG: Kyoto Encyclopedia of Genes and Genomes

We identified 1393 DEGs with a fold change |logFC|> 0.15 and an FDR < 0.05. After WGCNA, we further analysed 135 samples (five were outliers) and eventually identified 9 modules. From the heatmap of module-trait correlations, the green module, including 87 genes, was most highly related to the m6A cluster (Additional file 6: Fig. S5, Fig. 3a–d). To further explore module biological function, GO enrichment analyses were conducted, and the results are shown in Fig. 4a–c. The biological functions were mainly enriched in covalent chromatin modification, endomembrane system organization and protein localization to the Golgi apparatus. The results of KEGG analysis are shown in Fig. 4d.

### Hub gene screening and molecular subtypes

**Fig. 5:**
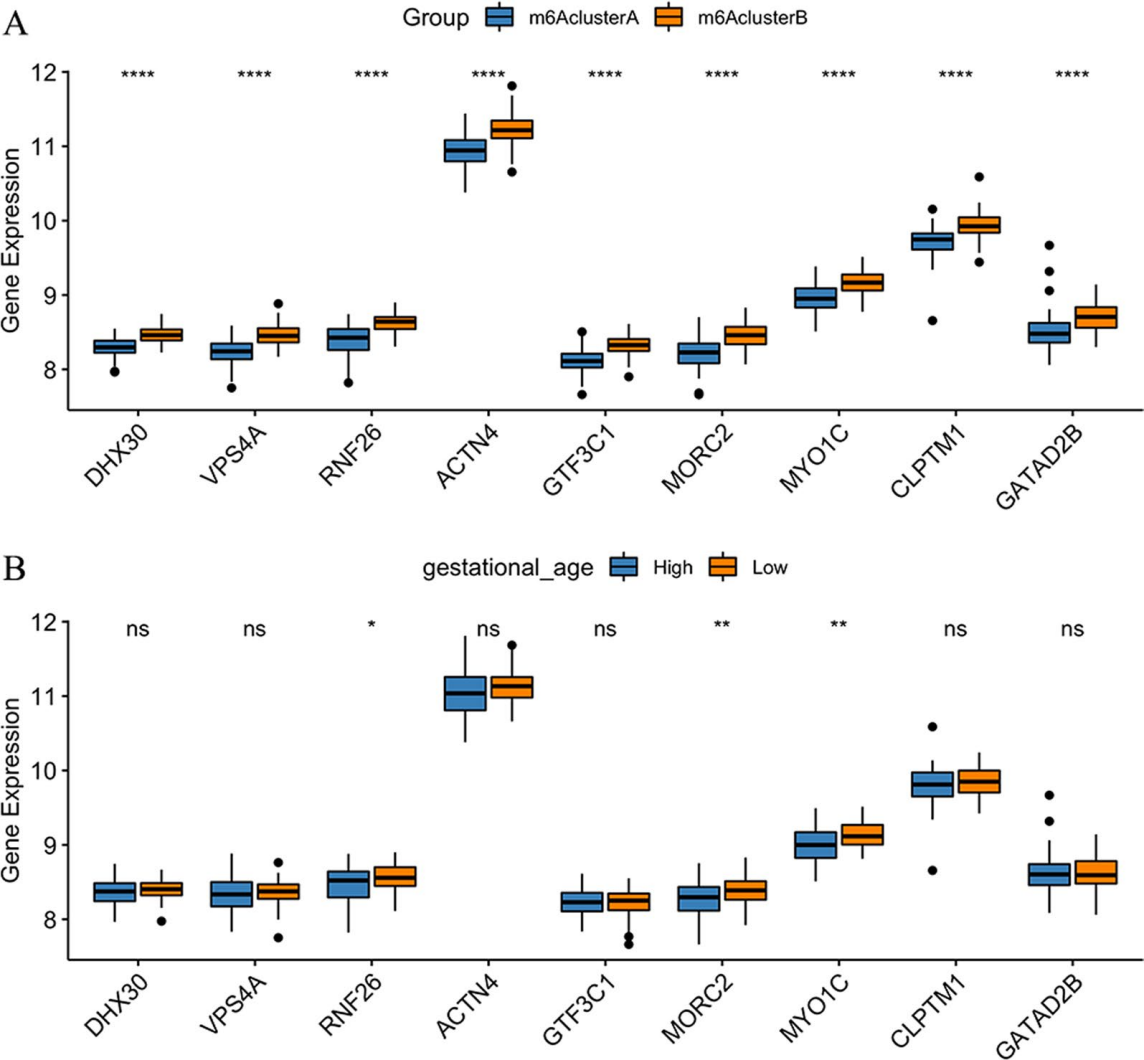
**a** Expression of nine hub genes in two distinct m6A clusters. **b** Expression of nine hub genes at different gestational age of patients with preeclampsia. The asterisks represented the statistical *P*-value (ns: *P* > 0.05; *P < 0.05; **P < 0.01; ***P < 0.001; ****P < 0.0001)

We further selected nine hub genes from the green module by setting MM > 0.7 and GS > 0.5. These hub genes included DHX30, VPS4A, RNF26, ACTN4, GTF3C1, MORC2, MYO1C, CLPTM1 and GATAD2B. We found that these nine hub genes were significantly different among the m6A clusters (Fig. 5a; *P* < 0.001). Furthermore, we explored the relationship between the expression of the nine hub genes and gestational age and found that the expression levels of RNF26, MORC2 and MYO1C were significantly associated with a low gestational age (Fig. 5b; *P* < 0.05).

**Fig. 6:**
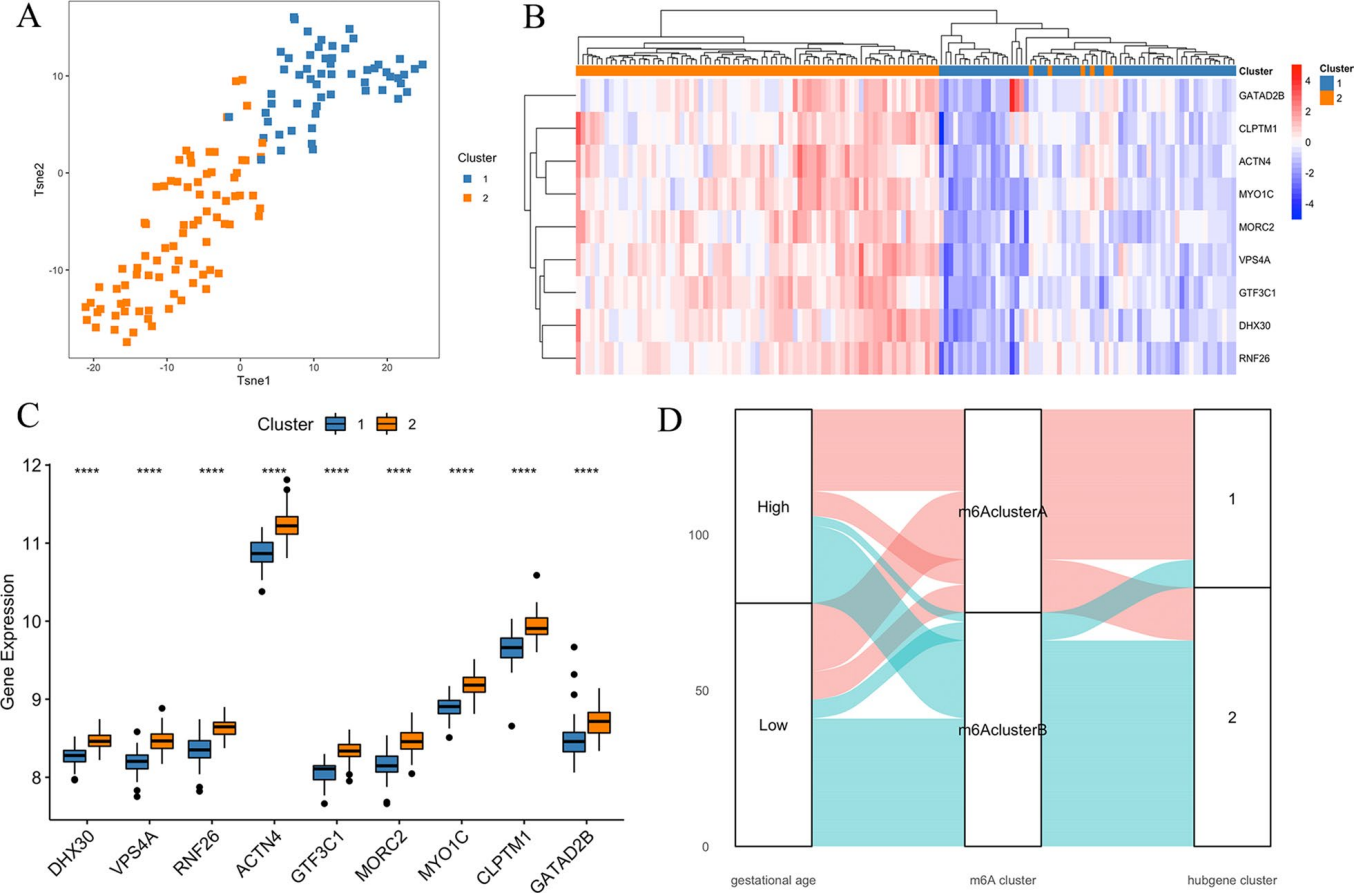
Different PE subgroups identified by unsupervised cluster analysis based on m6A hub gene expression. **a** Consensus clustering identified two gene clusters according to the expression of m6A regulators. **b**, **c** Heat map and violin diagram of levels of nine hub genes in cluster 1 and cluster 2; **d** Sankey diagram showing the relationship of gestational age, m6A clusters, and hubgene clusters. PE: preeclampsia

According to the results obtained, we used the K-means unsupervised clustering method to classify the PE samples (Additional file 6: Figure S5). As shown in Fig. 6a, the PE samples could be well divided into two clusters based on m6A hub gene expression. We found that the expression levels of the nine genes were significantly different between the two clusters, with lower gene expression in gene cluster 1 and higher gene expression in gene cluster 2 (*P* < 0.05, Fig. 6b, c), which could be regarded as signatures for different PE classification. For a better understanding of the association of m6A clusters, gestational age and hub gene clusters, we conducted a correlation analysis and found that almost all patients in m6A cluster A were classified into hub gene cluster 1. The expression of all nine m6A-related hub genes was significantly higher in gene cluster 1 than in cluster 2 (Fig. 6d). However, no significant difference in gestational age was observed between m6A cluster A and cluster B.

There are limited clinical data in GSE60438, while GSE75010 has more details. The common clinical features of these three sets include gestational weeks, infant sex and maternal age. We further analysed the differences in clinical features between the two PE groups. We found that when using 32 gestational weeks as a cut-off, cluster 1 had more patients with at least 32 gestational weeks (*P* = 0.086). There were no differences in infant sex or maternal age. Because GSE75010 has more details, we analysed the clinical characteristics of the 1-min Apgar score, the 5-min Apgar score, diagnosis of HELLP, diagnosis of intrauterine growth restriction (IUGR), maternal BMI, previous history of hypertensive pregnancy, miscarriage and nulliparity. We found that patients in cluster 1 were more likely to present with IUGR (*P* = 0.026).

## Discussion

PE is a unique hypertensive disorder in human pregnancy, and placental trophoblast dysfunction plays significant roles in the pathogenesis of this pregnancy disorder. The exact pathophysiology that causes PE remains unclear; however, genetic, immunological, endocrine, and environmental factors have been implicated in its pathogenesis [15]. The placenta plays an essential role in the development of PE. While the mechanism of abnormal placentation is controversial, animal models have demonstrated that uteroplacental ischaemia drives the hypertensive, multiorgan failure response observed in maternal preeclamptic syndrome [16]. Previous studies found increased m6A expression and increased m6A RNA methylation in placental trophoblasts in PE patients, suggesting that aberrant m6A RNA methylation may contribute to trophoblast dysfunction in this pregnancy disorder. Further determining whether increased m6A RNA methylation is associated with altered clinical features and identifying different subtypes according to m6A methylation levels will be helpful for understanding this disease.

In this study, we first analysed the expression of 21 m6A factors and identified the correlations between them. According to the expression levels of m6A, 140 samples were well divided into two groups. We then investigated the clinical characteristics of such groups and found that when using 32 as the cut-off for maternal age, cluster A was enriched in the high maternal age group (*P* = 0.011). Cluster A was also related to a high rate of IUGR (*P* = 0.055). The number of reports regarding the profiling of m6A modification and its potential role in the placenta of PE is small. Several studies have investigated the clinical features in PE patients and healthy controls and found no difference in maternal age in the whole group [17, 18]. Currently, no study has analysed the correlation between maternal age and m6A levels in PE patients, and our results need to be further explored.

Given the indispensable function of RNA m6A modification in various bioprocesses, it is reasonable to speculate that deregulation of m6A modification may also be associated with PE. Recent studies showed that METTL3 and METTL14 were upregulated in PE and verified that the significant increase in HSPA1A mRNA and protein expression was regulated by m6A modification, suggesting that m6A plays a key role in the regulation of gene expression [18]. Increased METTL3 expression and m6A RNA methylation were also observed by Taniguchi et al., and they suggested that aberrant m6A modification may contribute to trophoblast dysfunction in PE [17]. Furthermore, m6A at the 5’-UTR and nearby stop codon in placental mRNA may play important roles in foetal growth and PE through MeRIP-Seq conducted on human placentas obtained from mothers of infants of various birth weights [13]. For example, in the present study, infants in the high m6A expression group were more likely to have IUGR, indicating that m6A may affect foetal growth.

To identify the differences between the two m6A clusters, we analysed the infiltrating immune cells and found that several types of immune cells were correlated with m6A levels. PE is associated with chronic immune activation that leads to an increased production of inflammatory cytokines by proinflammatory T cells and a decrease in regulatory and anti-inflammatory cytokines, which further promotes an inflammatory state during PE [19, 20]. Macrophages, natural killer (NK) cells, dendritic cells (DCs), T cells, and T regulatory cells (Tregs) are present in the decidua and are required for the normal invasion of trophoblasts during placentation [21]. The Th1 and Th17 subclasses are responsible for promoting inflammation during PE, while Tregs and Th2 cells are decreased and are therefore unable to properly control the inflammation associated with increased inflammatory T-cell populations [22]. It has been proven that m6A methylation of mRNA controls T-cell homeostasis [23]. In the present study, Th17 and Th1 cells were enriched in m6A cluster A, which indicated that low levels of m6A promoted the activation of Th17 and Th1 cells.

Oxidative stress is regarded as an important immune event during PE and contributes to the development of inflammation in the vasculature, which includes the production of inflammatory IL-17 [24]. Another role of IL-17 that contributes to hypertension during pregnancy is stimulating B-cell production of agonistic autoantibody to the Ang II, type 1 receptor [25, 26]. Moreover, increased inflammatory cytokines, such as TNF-α, IL-6 and IL-17, are observed in the circulation of RUPP rats, while regulatory cytokines, IL-10 and IL-4, are decreased [27]. IL-10 and Tregs also improve the pathophysiology of PE through Th1 regulation and by exhibiting anti-inflammatory effects in general. m6A modification has been demonstrated to be a major posttranscriptional regulator of immune responses in cells [23]. The immune environment in PE is complex, and m6A regulation may contribute to the development of PE. Further evaluation of the correlation between m6A and infiltrating immune cells in PE is important.

We identified 1393 DEGs between the two m6A clusters, and then we performed WGCNA. According to the results, 87 hub genes were selected for further analysis. GO and KEGG analyses revealed that these DEGs were significantly enriched in different pathways and functions, including vasopressin-regulated water reabsorption, the AMPK signalling pathway, covalent chromatin modification, positive regulation of intracellular transport, and organelle localization. AMPK activation is required for placental differentiation and vasodilation of uterine artery blood flow [28]. Lack of AMPK induces malplacentation, which results in angiogenic imbalance. The increase in serum AMPK in severely preeclamptic women suggests a compensatory mechanism for the angiogenic imbalance [28]. AMPK activators ameliorate preeclamptic symptoms, which indicates that AMPK is a potential therapeutic target for PE. As described before, Treg and Th17 cells respond to the development of PE, and AMPK activation restores the normal balance between Treg and Th17 cells and cures such an imbalance [29]. Future studies are recommended to verify whether AMPK signalling may provide a prospective therapeutic target for the prevention and treatment of PE.

α-Actinins (ACTNs) are cytoskeletal proteins that maintain cytoskeleton integrity and control cell movement. Several studies have indicated that ACTN4 may participate in endothelial cell regulation. Moreover, recent studies have shown that ACTN4 is involved in cell apoptosis, which plays an important role in the progression of PE [30, 31]. Zhao et al. observed decreased expression of ACTN4 in severe PE endotheliocytes and demonstrated that dysregulated ACTN4 expression may be associated with PE due to its effects on endothelial cell apoptosis via the p38-MAPK/p53 apoptosis pathway [32]. GATAD2B expression was significantly decreased in pregnant mouse and human myometrium during labour. Chen et al. suggested that GATAD2B serves as an important mediator of P4–PR suppression of proinflammatory and contractile genes during pregnancy [32]. Other genes identified in this study may also be related to PE progression and have not been well investigated in PE. Microrchidia family CW-type zinc finger 1 (MORC1) is a highly conserved nuclear protein that is increasingly recognized as an epigenetic regulator [33]. MORC2 is a newly identified chromatin remodelling enzyme with an emerging role in the DNA damage response, but the underlying mechanism remains largely unknown [34]. RNF26 belongs to the RING domain family of proteins, which plays a key role in organizing the endosomal pathway for efficient cargo transport by mediating the ubiquitination of SQSTM1 [34]. Moreover, RNF26 has been reported to limit the type I interferon response by promoting the autophagic degradation of IRF3 [35]. All three genes were differentially expressed between the two gene groups and different maternal age groups in the current study. The correlation between PE and the nine hub genes, especially the potential methylation, needs to be further revealed.

Previous studies have illustrated gestational age as one of the important factors that influence methylation, and some studies have reported that women undergoing spontaneous preterm delivery may possess differences in methylation compared with those with an additional week of gestation for a total of at least 37 weeks [12, 36]. Furthermore, the augmented m6A levels at the 5’-UTR in mRNAs of small-for-date placental samples were dominant compared to the reduction in m6A levels, whereas a decrease in m6A in the vicinity of stop codons was common in heavy-for-date placental samples [13]. There is a lack of studies investigating whether there are some differences in the expression level of m6A at different PE gestational ages. According to our results, gestational age did not affect the m6A level, but when compared in different gene groups, the patients with high gene expression were enriched in gestational weeks less than 32 weeks. This indicated that gestational age is an important confounder when studying variation in placental DNA methylation. Fewer gestational weeks may be associated with more m6A-associated events.

There are some limitations in this study. First, due to the limited clinical information and the lack of foetal outcome data in GSE60438, the differences between two m6A clusters as well as gene clusters could not be well identified. Furthermore, studies focusing on m6A methylation in PE are limited, and some genes revealed in this study are newly recognized and have not been well researched in PE. It is difficult to explain the mechanism of such genes identified in this study.

## Conclusions

This is the first study to evaluate the m6A level and potential related genes in PE patients. Our results revealed that a high m6A level is associated with lower maternal age and IUGR occurrence. Several immune cells may contribute to different m6A clusters and clinical features. Furthermore, nine hub genes that mediated classification could well reflect the m6A level. The high hub gene expression group is correlated with a high IUGR occurrence rate and gestational weeks. Our results indicated that the m6A level plays an essential role in PE development and outcome for both the mother and foetus. Currently, there is no gene risk model for PE patients. The nine hub gene model established in this study reflected that different m6A levels can be used to well identify patients at high risk for PE.

## Supplementary Information

**Additional file 1: Table S1.**The number of genes in each module.

**Additional file 2: Fig. S1.**Heatmap of the matrix of co-occurrence proportions for preeclampsia samples.

**Additional file 3: Fig. S2.**Consensus clustering cumulative distribution function for k = 2–6.

**Additional file 4: Fig. S3.**Evaluation of the pairwise correlations among 17 m6A regulators’ expression.

**Additional file 5: Fig. S4.**Difference in maternal age between two m6A clusters.

**Additional file 6: Fig. S5.**Analysis of the scale-free ft index and the mean connectivity for various soft-thresholding powers.

## Data Availability

Microarray datasets (GSE75010 and GSE60438) for this study are openly available in Gene Expression Omnibus database at https://www.ncbi.nlm.nih.gov/geo/query/acc.cgi?acc=GSE75010 and https://www.ncbi.nlm.nih.gov/geo/query/acc.cgi?acc=GSE60438, respectively.

## Abbreviations

PE: Preeclampsia
m6A: N6-methyladenosine
m5C: 5-Methylcytosine
m1A: N1-methyladenosine
UTR: Untranslated region
GEO: Gene expression omnibus
PCA: Principal component analysis
ssGSEA: Single-sample gene-set enrichment analysis
DEGs: Differentially expressed genes
FDR: False discovery rate
FC: Fold change
WGCNA: Weighted gene coexpression network analysis
TOM: Topological overlap matrix
MEs: Minimum module eigengenes
GS: Gene significance
KEGG: Kyoto encyclopedia of genes and genomes
GO: Gene ontology
MM: Module membership
t-SNE: T-distributed stochastic neighbour embedding
IUGR: Intrauterine growth restriction
NK: Natural killer
DCs: Dendritic cells
Tregs: T regulatory cells
ACTNs: α-Actinins
MORC1: Microrchidia family CW-type zinc finger 1
