# Supplementary figures and images for: Gene model-related m6A expression levels predict the risk of preeclampsia
Source: BMC Med Genomics. 2022 May 5;15:103. doi: 10.1186/s12920-022-01254-4 (PMC9069853; doi:10.1186/s12920-022-01254-4)

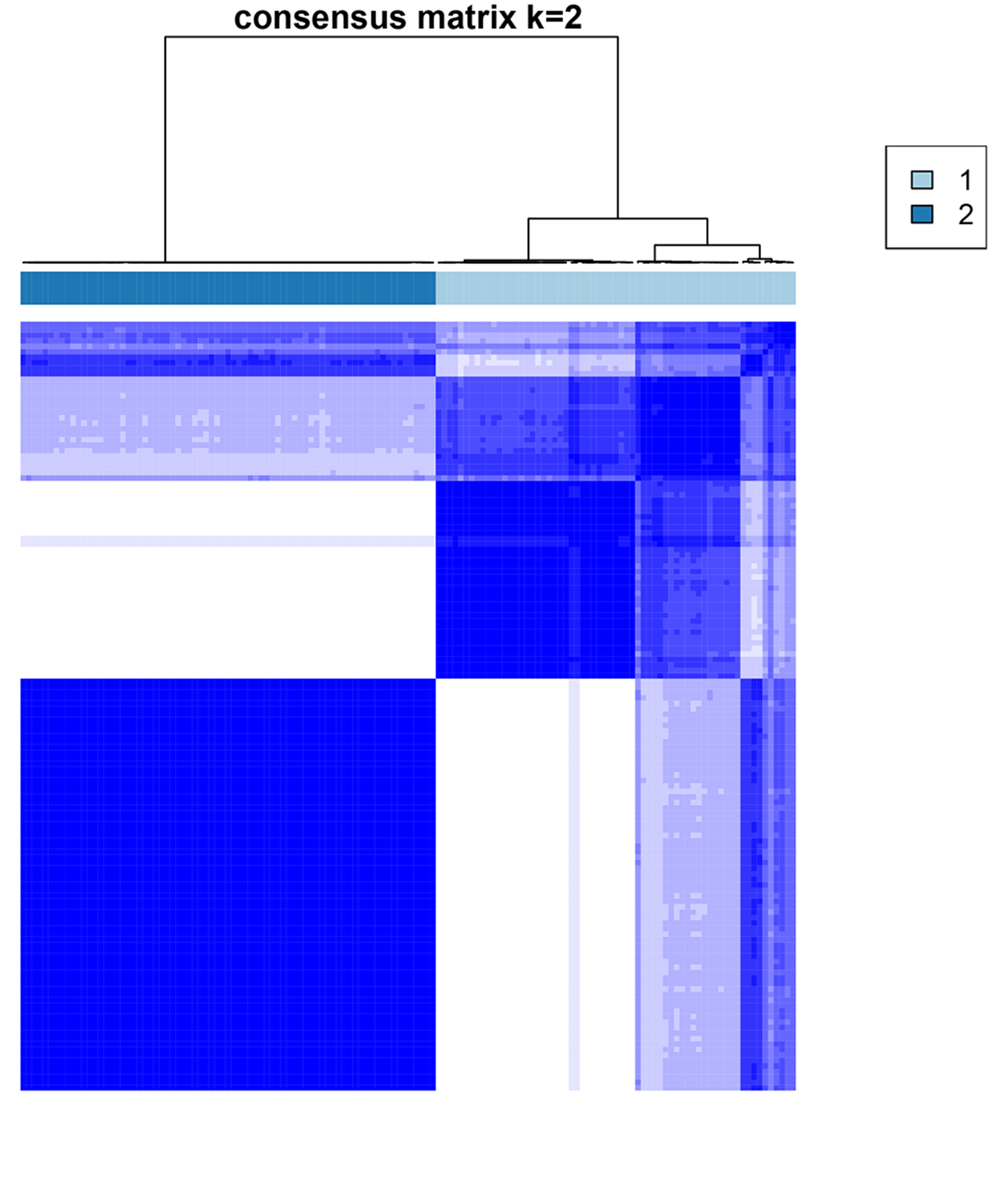

Supplement: Supplementary file 2 — Additional file 2: Fig. S1.Heatmap of the matrix of co-occurrence proportions for preeclampsia samples. [file 12920_2022_1254_MOESM2_ESM.tif]

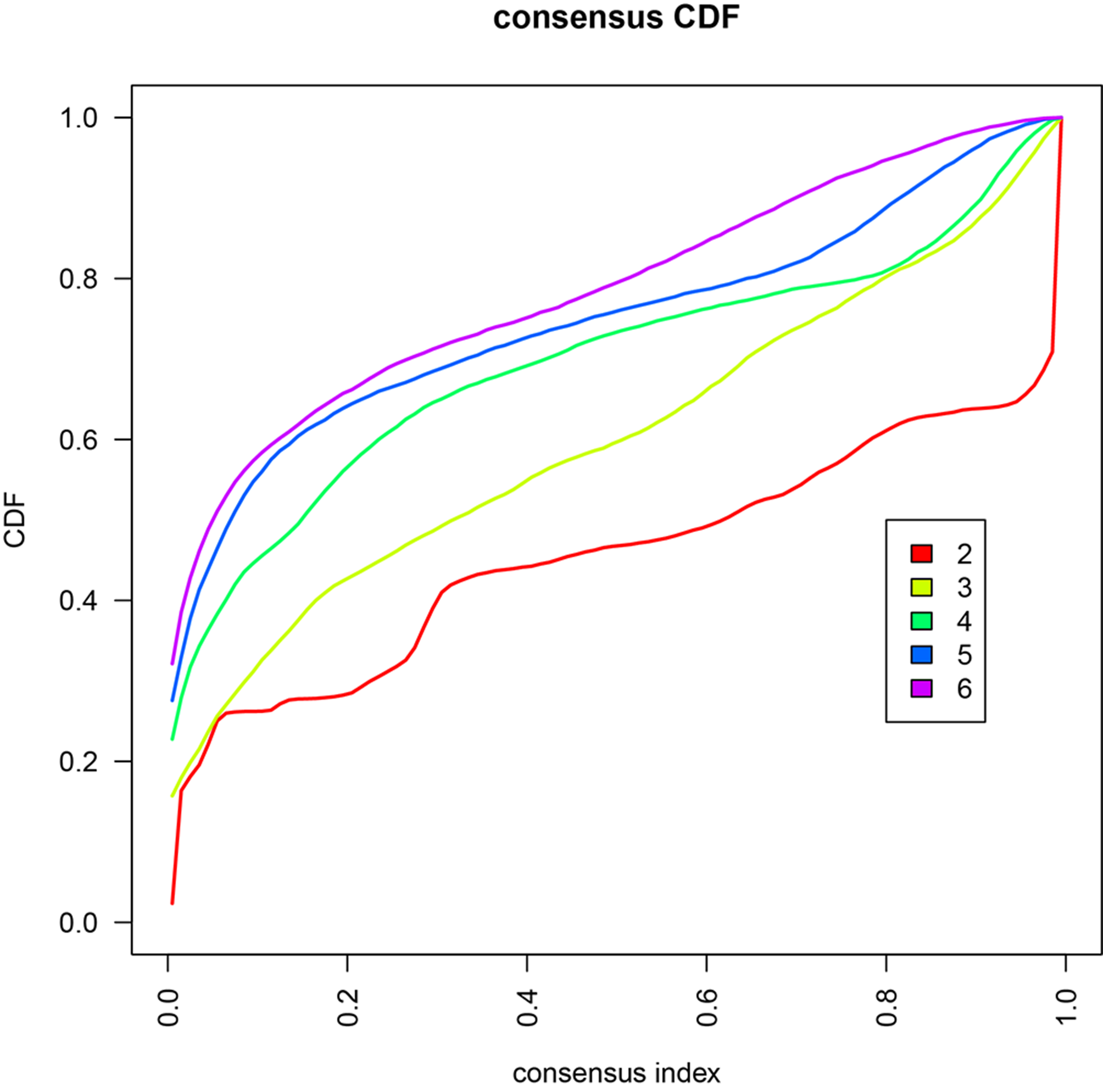

Supplement: Supplementary file 3 — Additional file 3: Fig. S2.Consensus clustering cumulative distribution function for k = 2–6. [file 12920_2022_1254_MOESM3_ESM.tif]

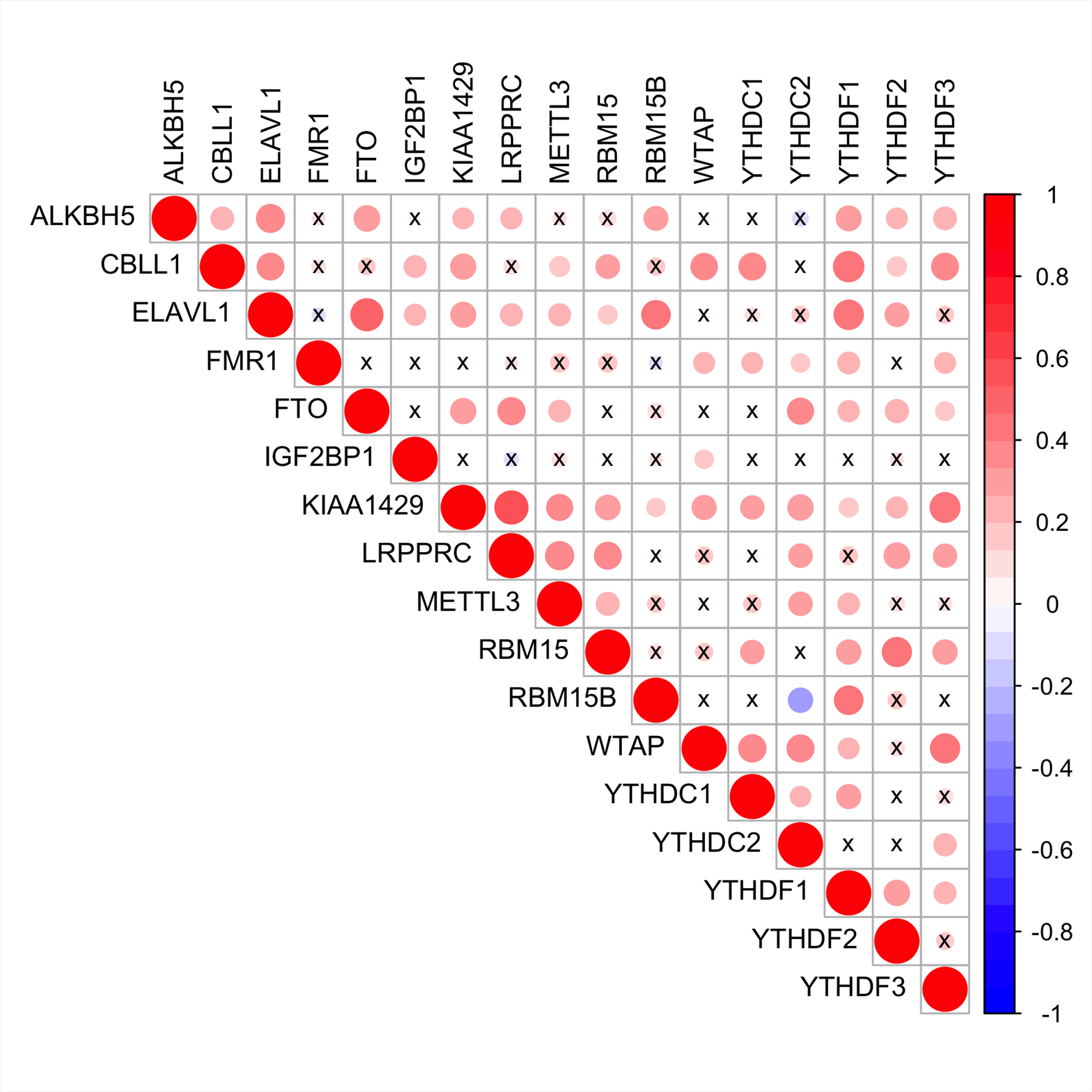

Supplement: Supplementary file 4 — Additional file 4: Fig. S3.Evaluation of the pairwise correlations among 17 m6A regulators’ expression. [file 12920_2022_1254_MOESM4_ESM.tif]

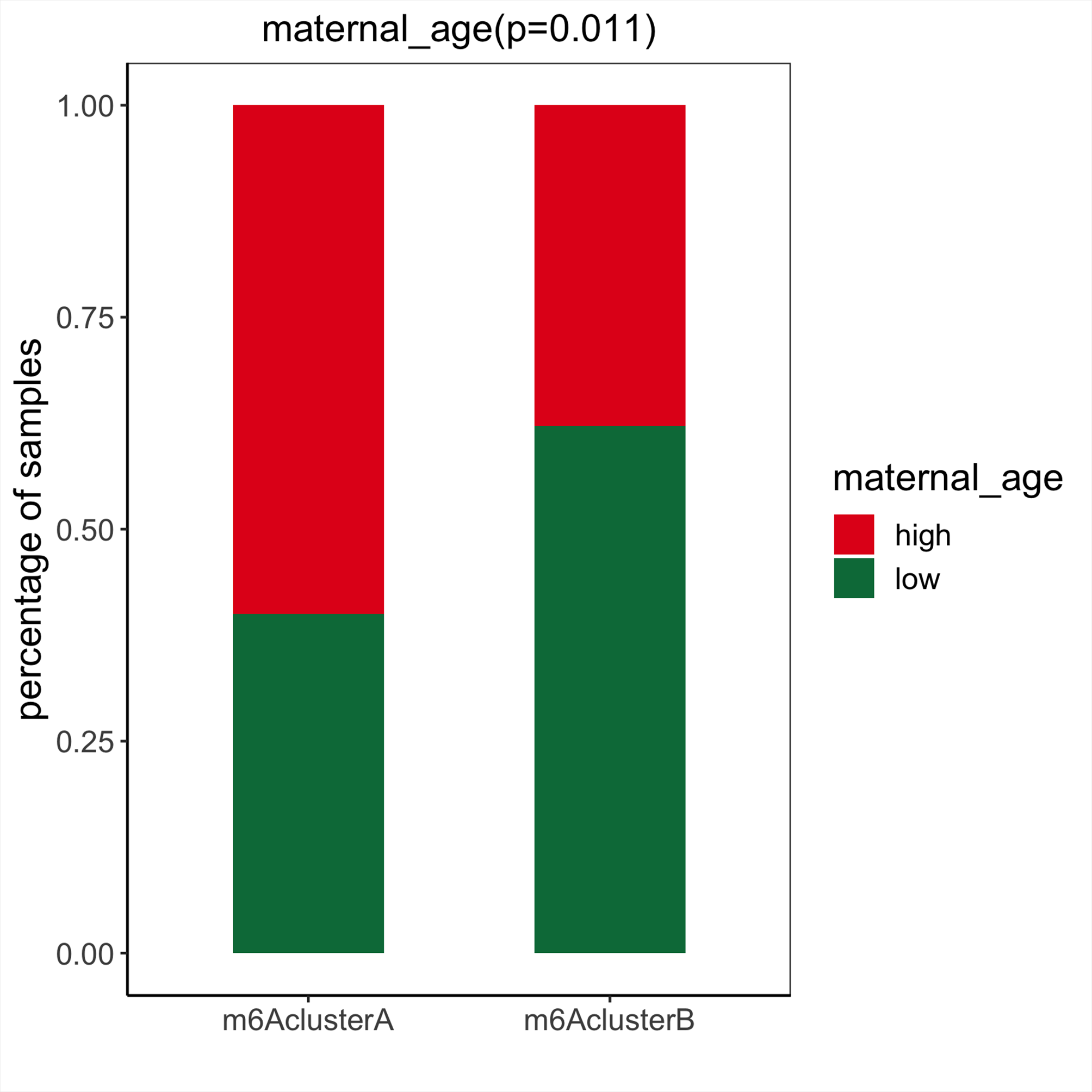

Supplement: Supplementary file 5 — Additional file 5: Fig. S4.Difference in maternal age between two m6A clusters. [file 12920_2022_1254_MOESM5_ESM.tif]

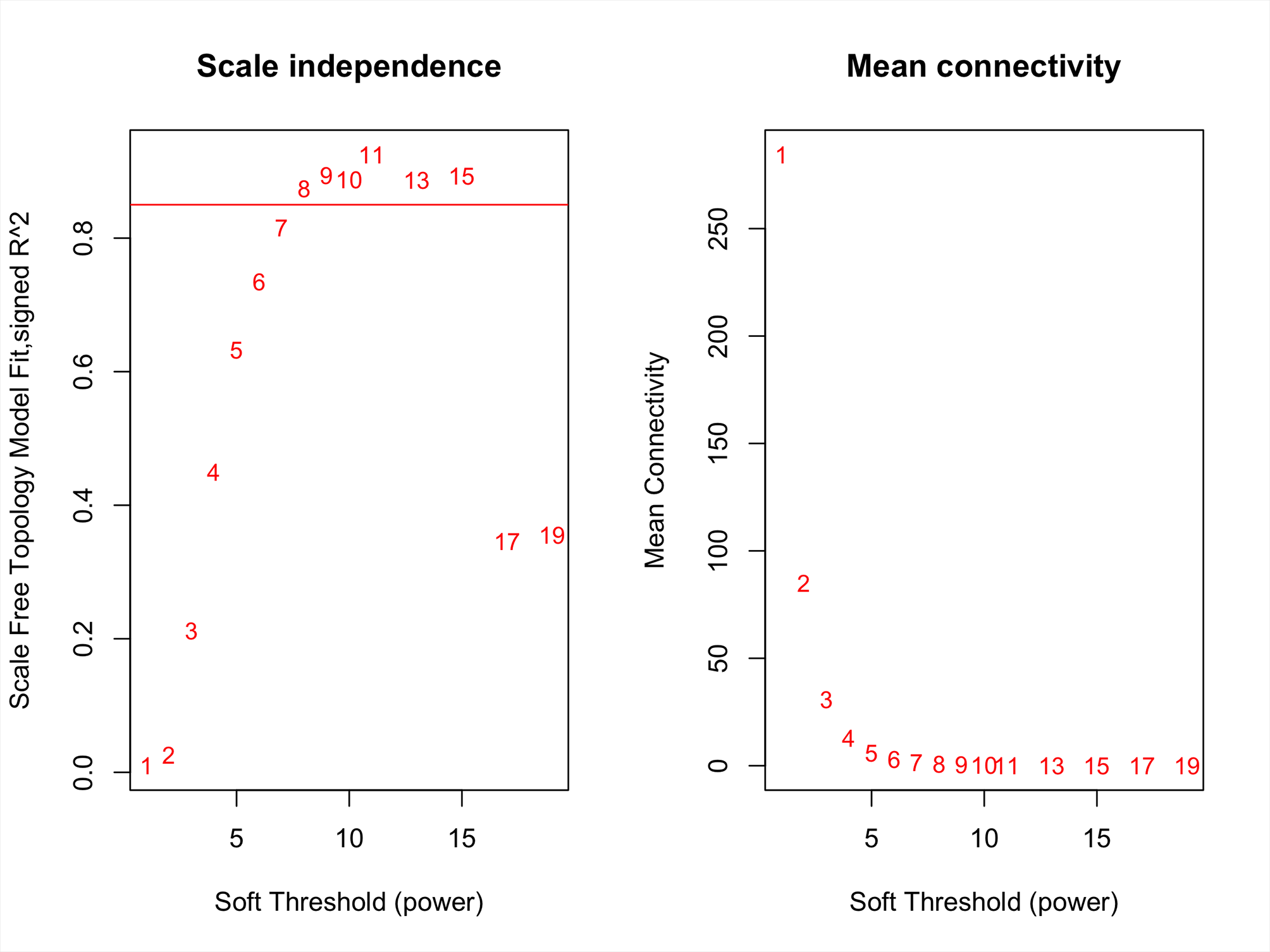

Supplement: Supplementary file 6 — Additional file 6: Fig. S5.Analysis of the scale-free ft index and the mean connectivity for various soft-thresholding powers. [file 12920_2022_1254_MOESM6_ESM.tif]
